# Effects of a Myofascial Technique on the Stiffness and Thickness of the Thoracolumbar Fascia and Lumbar Erector Spinae Muscles in Adults with Chronic Low Back Pain: A Randomized before-and-after Experimental Study

**DOI:** 10.3390/bioengineering10030332

**Published:** 2023-03-06

**Authors:** Karine Devantéry, Mélanie Morin, Julien Grimard, Nathaly Gaudreault

**Affiliations:** Faculty of Medicine and Health Sciences, School of Rehabilitation, University of Sherbrooke, 3001 12th Avenue North, Sherbrooke, QC J1H 5N4, Canada

**Keywords:** sonoelastography, ultrasonography, low back pain, thoracolumbar fascia, myofascial release, manual therapy, osteopathy

## Abstract

The thoracolumbar fascia (TLF) may be a pain generator, given its rich innervation. Structural and biomechanical changes have also been documented in adults with chronic non-specific low back pain (LBP). Myofascial techniques (MFTs) are commonly used in manual therapy and are hypothesized to reduce tissue stiffness and pain. However, evidence for these effects is limited. The objective of this study was to evaluate the immediate effects of a standardized MFT compared to a simulated MFT on: (1) the stiffness of the TLF and erector spinae muscles (shear-wave sonoelastography), (2) the thickness of the TLF (B-mode ultrasound), and (3) pain intensity (numerical rating scale). Forty-nine participants with chronic non-specific LBP were included in a randomized before-and-after experimental study. Outcome measures were collected before (T0) and immediately after the intervention (T1). Pain intensity was also assessed on day two (T2) and seven (T7). The MFT group showed a significant decrease in left erector spinae muscle stiffness and left TLF thickness compared to the simulated group. In addition, there was a significant reduction in pain intensity in the MFT group compared to the simulated group at T1 and T2. The results of this study suggest that MFT results in immediate tissue changes and transient pain reduction in patients with LBP.

## 1. Introduction

Low back pain (LBP) is a worldwide health issue with an estimated average point prevalence of 32.9% and a lifetime prevalence of up to 84% in high-income countries [1,2]. As the leading cause of years lived with a disability [3,4], LBP has major consequences for quality of life [5] and results in significant financial burden [6]. Ninety percent of LBP is classified as non-specific, where the pain is not attributed to a known identifiable specific pathology [2,7]. An increasing number of studies have reported that fasciae may play a role in chronic pain, particularly in some unexplained LBP conditions [8,9,10,11,12,13,14], due to their rich nociceptive innervation [15,16,17,18,19,20,21].

The thoracolumbar fascia (TLF) lies beneath the deep adipose tissue. It is an aponeurotic fascia composed of three layers of densely packed collagen and elastic fibers, separated by loose connective tissue containing hyaluronan [22,23]. Hyaluronan acts as a lubricant allowing the layers to glide over each other, which is essential for musculoskeletal mobility [13,23,24,25]. The epimysium of the erector spinae muscle lies underneath the posterior layer of the TLF, from which it is separated by a layer of loose connective tissue [23]. Owing to recent technological advancements in ultrasound imaging, the structure and mechanical behaviors of the TLF and erector spinae muscles of adults presenting chronic LBP can now be evaluated non-invasively. Studies have shown that patients with LBP have (1) a 25% increase in TLF thickness [9]; (2) a 20% decrease in TLF shear strain [10]; (3) a 20% increase in erector spinae muscle stiffness [26]. These changes could be explained by fibrosis and adhesions developing as a result of local connective tissue inflammation and/or injury and decreased mobility due to pain. Among other things, these tissue alterations could, in turn, act as predisposing factors for LBP or chronicity factors [8,9,10,11,27,28].

Over the last three decades, there has been a proliferation of different forms of myofascial therapy (MFT) targeting fasciae. The general goals of these techniques are to decrease tension in the myofascial tissues, increase function, and reduce pain [29]. Recently, two systematic reviews concluded that MFTs lead to a decrease in disability in patients presenting LBP [30,31], and confirmed the effect in reducing pain intensity [31]. These studies are of interest but do not report the specific effects of MFTs. As MFTs are supposed to target fasciae, an evaluation of the changes in the structure and biomechanical properties of the lumbar fasciae could provide information about the specific effects of these techniques and allow a better understanding of the underlying mechanisms of MFTs.

A few studies have already investigated the effects of MFTs on the structure and biomechanical properties of fasciae using high-performance imaging techniques, such as ultrasonography or sonoelastography, and have shown promising results [32,33,34]. The structural and biomechanical effects reported are a reduction in the thickness of the fasciae of the neck muscles [32], an increase in fascial mobility of the vastus lateralis muscle [34], and a decrease in stiffness in the posterior layer of the TLF [34]. Other studies have focused on the effects of MFTs on muscle and observed a reduction in iliocostalis and erector spinae muscle stiffness [35], an increase in muscle displacement [36] and a reduction in the elastic modulus of the erector spinae [37].

The results of these studies are interesting but limited. The only study specifically addressing the TLF was conducted on a small sample (n = 10) of asymptomatic participants with no control intervention [34]. In addition, only two of the three studies on the lumbar muscles specifically evaluated the effects of MFTs, but used indirect stiffness assessment techniques [36,37]. Evidence for the specific effect of MFTs remains to be established.

The main objective of this study was to evaluate the immediate effect of a standardized versus a simulated MFT on the stiffness of the TLF and lumbar erector spinae muscles using shear wave elastography. The secondary objectives were to evaluate the immediate effects of the MFT versus the simulated MFT on (1) the thickness of the TLF and (2) pain intensity. We hypothesized that the standardized MFT would lead to a decrease in TLF and erector spinae muscle stiffness, a decrease in the thickness of the posterior layer of the TLF, and an immediate/short-term decrease in pain intensity when compared with the simulated MFT.

## 2. Materials and Methods

### 2.1. Design and Setting

A before-and-after experimental study was carried out at the Research Center of the *Centre Hospitalier Universitaire de Sherbrooke* (CHUS) in Quebec, Canada, from March to July 2021. The study was approved by the Ethics Committee of the *Centre intégré universitaire de santé et de services sociaux de l’Estrie-CHUS* in Canada (CIUSSS-Estrie CHUS, 2021–3572) and was registered at ClinicalTrials.gov (NCT04830566).

### 2.2. Participants

A total of 49 participants were recruited through advertisements on the *Université de Sherbrooke* campus, rehabilitation/osteopathic clinics in the Sherbrooke area, and social media, as well as by word of mouth. To be included in the study, participants were required to (1) be adults over the age of 18; (2) have pain localized between the 12th rib and the gluteal fold for more than six months; (3) have back pain intensity >3/10 on the numerical rating scale (NRS); (4) suffer from back pain for more than 50% of the time during the day. Participants were excluded if they (1) had a history of serious spinal or lower-extremity injury or a history of surgery in the past year; (2) had back pain due to a known pathology or symptoms reflecting a possible pathology; (3) received oral or injectable corticosteroids in the lumbar spine in the last three months; (4) had a psychiatric disorder; (5) were pregnant; or (6) received manual therapy applied to the lumbosacral region in the month preceding the intervention and during the course of the study. All participants signed a written informed consent form prior to enrollment.

### 2.3. Randomization and Blinding

The participants were randomly assigned to one of the two groups (1:1; random block size 2–4). Given the differences in male/female TLF thickness and stiffness reported in the literature [9,38], randomization was stratified by gender. To ensure randomization concealment, a research assistant that was not involved in the study generated the randomization list and prepared envelopes containing the group allocation of each participant. The envelopes were opened by the researcher at treatment time. Participants were blinded to the group to which they were assigned. The assessments of tissue stiffness and thickness were performed by a blinded physiotherapist. The research assistant in charge of extracting the ultrasound and elastographic data was also blinded to both the intervention received and the measurement time. The data on pain intensity at all time points were collected by the osteopath who performed the intervention.

### 2.4. Procedures

**Figure 1:**
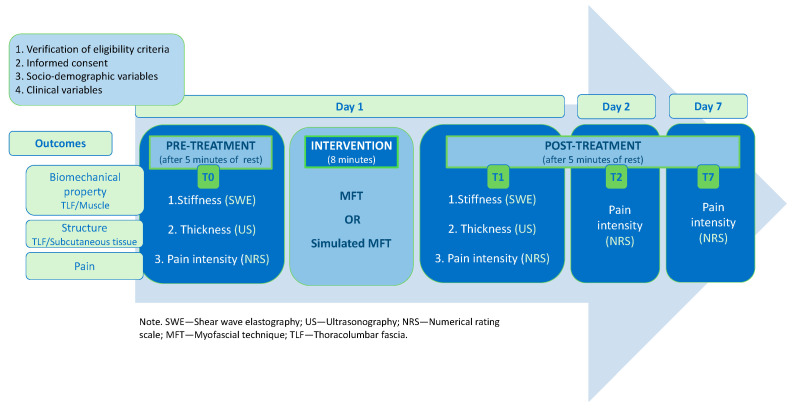
Study timeline.

The timeline of the study is presented in Figure 1. Participants were convened at a pre-treatment assessment (T0) conducted by a physiotherapist, who was trained and experienced in musculoskeletal ultrasound (8 years of experience) but not involved in the treatments and, therefore, blinded to group allocation. Sociodemographic and clinical data were collected, including body mass index, hand dominance, duration of LBP, lumbar flexion range of motion (Modified Schober Index), pain quality and intensity (Short-form McGill Pain Questionnaire/NRS), impact of pain on function (Oswestry Disability Scale), and physical activity level (International Physical Activity Questionnaire). The participants were then asked to adopt a prone position with their head in a neutral position, a pillow under their legs to reduce tension on their lower back, and a towel under their shoulders for comfort. The L2–L3 interspinous space was localized by manual palpation and a mark was made on the skin at this level. The precision of this landmark was verified using B-mode ultrasound and corrections were made whenever necessary. The probe was then placed 2 cm lateral to this mark, on the right and left sides, and its contour was drawn on the skin on both sides with a permanent marker to ensure that the probe would be at the same location and in the same position for each measurement. The participants were then asked to rest for five minutes without moving. After the resting period, sonoelastographic measurements were taken on the left and right sides with state-of-the-art instruments and validated procedures [9,10,39]. All measurements were taken at the end of exhalation by asking the participants to briefly hold their breath to limit breathing movements [40]. After these measurements, an independent osteopath with over 10 years of experience applied either the MFT or the simulated MFT according to group allocation. After the intervention, participants were asked to rest in the same position without moving for five minutes to ensure the uniformity of the evaluations, as resting can lead to an increase in myofascial stiffness [41]. Thereafter, a blinded evaluator conducted the post-treatment assessment (T1) comprising the sonoelastographic evaluation. Pain measurements (NRS) were performed before treatment (T0), immediately after treatment (T1), and 24 h (T2) and one week post-treatment (T7).

### 2.5. Treatment

**Figure 2:**
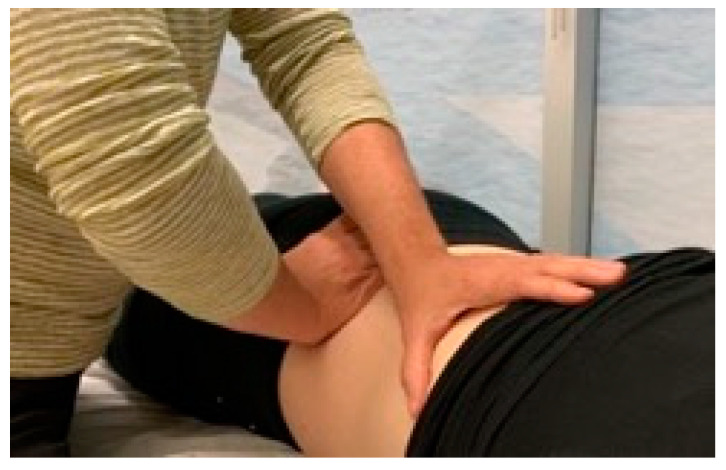
Hand positioning for the techniques.

An experienced osteopath performed both the standardized and simulated MFTs. The MFT was a direct myofascial release technique. According to the definition in the glossary of osteopathic terminology, “direct myofascial release” is a method that uses continual palpatory feedback where “the dysfunctional myofascial tissues are loaded and the restrictive barrier is engaged with a constant force” [42] (p. 38). The MFT was performed as in routine clinical practice. The osteopath was positioned laterally to the participant, with their forearms crossed, caudal hand on the ilium, and cephalad hand on the posterior/inferior thorax, as described by Wong et al. [34] (Figure 2). The cephalad hand was sometimes moved towards the center or the contralateral thorax depending on the perceived tension. Gentle pressure was applied in order to target the TLF and erector spinae muscles. The therapist then applied tension between her hands in the different planes, according to the perceived tension, until reaching a restrictive barrier. The tension was maintained until an initial release was perceived; then, the therapist adjusted the tension applied on the tissue until reaching a new restrictive barrier. The technique was performed for four minutes on each side, starting on the left. The simulated MFT was performed in the same way but with superficial contact on the skin and no tension applied between the hands.

### 2.6. Outcomes and Instruments

#### 2.6.1. Primary Outcome—Stiffness

The stiffness of the TLF and erector spinae muscles was evaluated using an ultrasound imaging device in shear-wave elastography (SWE) mode (Aixplorer Ultimate, SuperSonic Imagine, Aix-en-Provence, France; SL 10-2 MHz linear probe). The ultrasound SWE mechanism involves a focal acoustic radiation force, which is transmitted to the tissue by a primary linear US array to produce local stress and displacement in the tissue [43]. Shear waves perpendicular to the initial focal wave then propagate through the adjacent tissues in the transverse plane, but at a slower velocity, causing shear displacement in the tissue. It is possible to calculate the shear wave velocity using a speckle tracking algorithm. The distribution of shear-wave velocities at each pixel is directly related to the shear modulus, which is expressed as Young’s modulus in kilopascals (kPa) by the Aixplorer. The shear modulus is strongly correlated with Young’s modulus [44,45], which is recognized as a measure of the intrinsic stiffness of a tissue. A stiffer tissue will produce a higher transmission rate and shear modulus [43]. A good to excellent intra- and inter-evaluator reliability of SWE stiffness measurements has been reported for the TLF [46] and erector spinae muscles [47]. SWE has been used in interventional studies [35,46], where the minimal detectable change in TLF was found to be 4.71 kPa in the sitting position [46].

**Figure 3:**
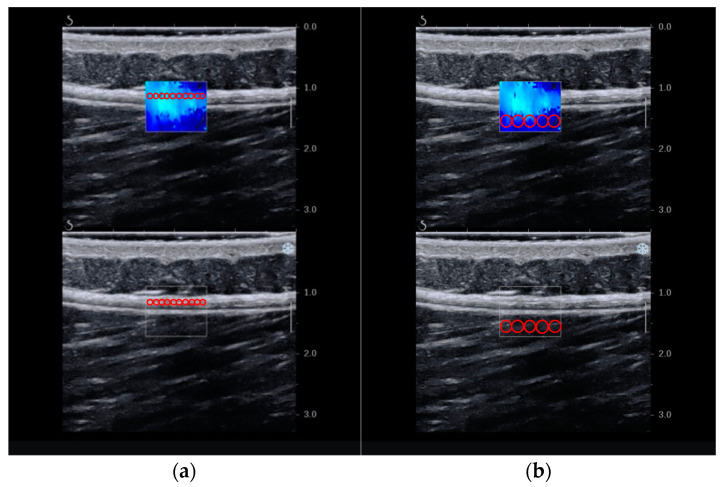
Regions of interest in elastographic images: (**a**) TLF and (**b**) erector spinae muscles.

In the present study, the probe was positioned parallel to the structures along the long axis and placed 2 cm laterally to the interspinous ligament, at the level of the L2-L3 interspinous space [9]. This location was proposed by Langevin et al. (2009), as it is an area where the fascia is parallel to the skin [9], allowing for optimal image acquisition. The evaluator made sure (1) to position the probe directly on the landmarks previously drawn on the skin laterally to L2-L3, which limited the possibility of rotational and translational displacements at different measurement times, and (2) to maintain the orientation of the probe so that the sound beam was perpendicular to the TLF to ensure homogeneity in the measurements and avoid artifacts. Prior to the study, intra-operator reliability was verified on 10 volunteers with different morphologies (20 images) and was shown to be good to excellent. During image acquisition, the elasticity map (SWE measurement box superimposed on the B-mode ultrasound image) was positioned centrally in the middle of the image, between the lower quarter of the deep adipose tissue layer and the superficial border of the erector spinae muscles (1 cm wide by 0.5 cm deep, see Figure 3). A total of three images were captured on the left and right sides, while ensuring that the coupling gel was visible in the ultrasound image in order to prevent tissue compression and, consequently, an overestimation of stiffness. The images were anonymized and analyzed retrospectively to ensure that the research assistant extracting the data was blinded to the participants and measurement times. In each image, he positioned 10 circles of 1 mm diameter on the middle of the TLF, ensuring that most of the length of the TLF within the elasticity map was covered (Figure 3a). The stiffness was calculated on the portion delineated by all 10 circles, called regions of interest (ROIs). For the erector spinae muscle, 5 circles of 2 mm were positioned 2 mm under the epimysium (Figure 3b). The US machine built-in software was used to calculate the median stiffness of the ROIs. The means of the three images were used for statistical analysis. Inter-evaluator reliability between the research assistant in charge of the extraction and an expert was conducted on 69 images, and the correlation was excellent both for the TLF and muscle stiffness (r > 0.999).

#### 2.6.2. Secondary Outcome—Thickness

**Figure 4:**
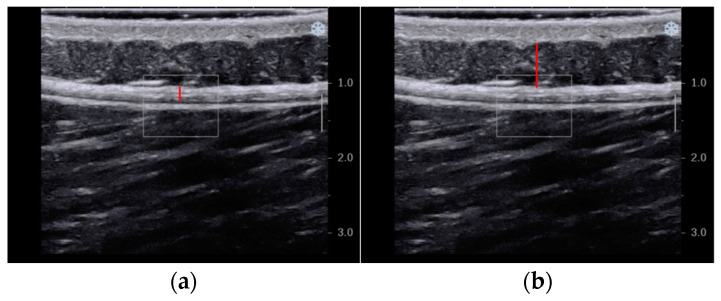
Thickness of the tissues in ultrasound images: (**a**) TLF and (**b**) subcutaneous tissue.

The structure of the fasciae was characterized by the thickness of the TLF. Since the thickness of the subcutaneous tissue may influence the propagation of the shear wave [43], this confounding variable was also measured. The subcutaneous tissue includes the superficial and deep adipose tissue [48]. The B-mode images captured for the primary outcome were used to calculate the thickness of both tissues. Measurements were made perpendicular to the longitudinal axis of the TLF (Figure 4). For both tissues, the mean of the three measurements on each side was used for statistical analyses. Ultrasound has previously been shown to have good inter-evaluator reliability (>0.98) for the measurement of lumbar perimuscular tissue thickness [9].

#### 2.6.3. Secondary Outcome—Pain Intensity

Pain intensity was assessed using the numerical rating scale (NRS; ranging from 0 to 10). The NRS was chosen because it is widely used in both clinical and research settings and has shown good sensitivity to change [49,50]. The participants were contacted by phone for measurements at T2 and T7. In the literature, a 20% change is considered clinically significant for chronic pain [49].

### 2.7. Statistical Analysis

A sample size of 48 participants was calculated prior to the beginning of the study, based on the results of Gao et al. [35], to detect a between-group difference in the change in stiffness, which was the main outcome (δ = 0.25; σ = 0.3; effect size = 0.8; power = 80%; alpha = 0.05%). The sociodemographic and clinical variables of the two groups were compared at pre-treatment using Fisher’s exact test, a Mann–Whitney U test, or an independent Student’s t-test according to the type of variable (categorial, ordinal or continuous) and the normality of the distribution. Generalized estimating equation models were used to assess and compare changes in stiffness, thickness, and pain outcomes in both groups (variable GROUP) over time (variable TIME). Treatment effects were assessed based on a significant GROUP*TIME interaction. This model was selected to account for the non-parametric distribution and imbalance at pre-treatment that could occur with a small sample size despite randomization. The proportions of participants in both groups with clinically meaningful changes in pain intensity (i.e., ≥2/10 [49]) were compared using a Chi-squared test. Statistical analyses were conducted at a level of significance of 0.05 using IBM SPSS Statistics (version 28. Armonk, NY, USA: IBM corp) and R statistics (version 4.0.2. Vienna, Austria: R Core Team).

## 3. Results

**Figure 5:**
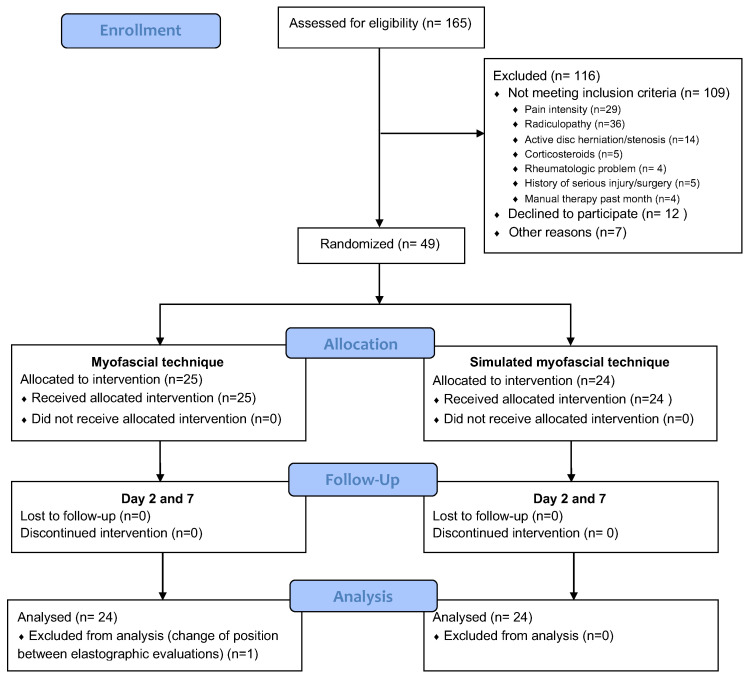
Participant flow diagram.

A total of 49 participants met the eligibility criteria and were enrolled in the study (25 male/24 female). They were randomized to the MFT group (intervention) or the simulated group (control). One participant was excluded from the analysis due to a change in position during experimentation, which is why one additional participant was included in the sample. Figure 5 shows the participant flow diagram.

### 3.1. Participants

The pre-treatment characteristics of the participants are presented in Table 1. A non-significant difference was found between the two groups for age, sex, duration of LBP, body mass index, lumbar range of motion (Modified Schober Index), pain quality (Short-form McGill Pain Questionnaire), impact of pain on function (Oswestry Disability Scale), and physical activity level (International Physical Activity Questionnaire).

### 3.2. Stiffness

Table 2 presents the stiffness data assessed with SWE at each time point and the mean differences between treatment groups. A significant difference between groups was found for changes in left erector spinae muscle stiffness (mean difference between groups: −16.1 kPa; 95% confidence interval (CI): −31.1 to −1.1; p = 0.035). The MFT group showed a reduction in left erector spinae muscle stiffness after treatment (from T0 54.0 ± 37.8 kPa to T1 41.5 ± 17.7 kPa), while the simulated group exhibited an increase in stiffness (from T0 39.5 18.2 kPa to T1: 43.1 ± 14.6 kPa). No significant differences between groups were observed in TLF or right erector spinae muscle stiffness.

### 3.3. Thickness

Table 3 presents tissue thickness data assessed with ultrasound at each time point and the mean differences between treatment groups. There was a significant difference between groups for changes in the thickness of the left TLF (mean difference between groups: −0.07 mm; 95% CI: −0.13 to 0.00; *p* = 0.039). For the left TLF, there was a very slight decrease in thickness in the MFT group (from T0 1.90 ± 0.50 mm to T1 1.88 ± 0.52 mm), while the simulated group showed an increase in thickness (from T0 1.78 ± 0.42 mm to T1 1.83 ± 0.43 mm). A significant between-group difference was observed for changes in the thickness of the right and left subcutaneous tissue (*p* < 0.001 and *p* = 0.007, respectively). There was an increase in thickness in the MFT group, while the simulated group showed a decrease in thickness (mean difference between groups right side: 1.15 mm; 95% CI: 0.6 to 1.71; mean difference between groups left side: 1.46 mm; 95% CI: 0.41 to 2.52). 

### 3.4. Pain Intensity

**Figure 6:**
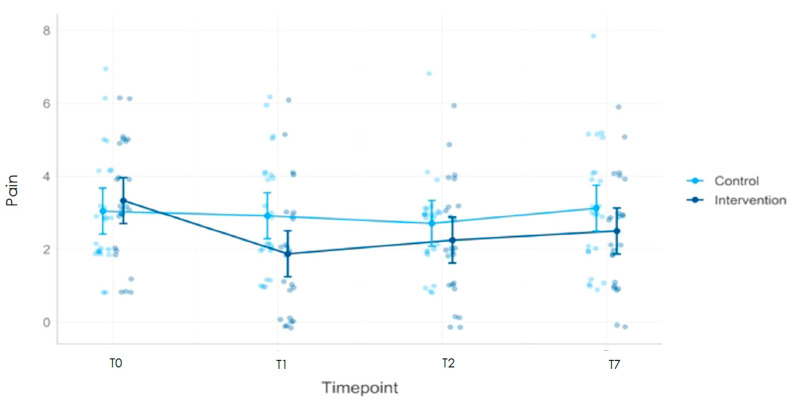
Evolution of pain intensity over time in the MFT group (intervention) and the simulated MFT group (control) using the numerical rating scale.

Table 4 and Figure 6 present the results for pain intensity assessed using the NRS at each time point and between treatment groups. There was a significant difference in the change in pain intensity between groups from T0 to T1 (mean difference between groups: −1.3; 95% CI: −2.1 to 0.6; *p* < 0.0001) and T2 (mean difference between groups: −0.8; 95% CI: −1.5 to 0.0; *p* = 0.043). The MFT group showed a significant decrease in pain intensity immediately after treatment, which was still significant 24 h later (T2). There was no significant change in pain intensity in the simulated group. Significantly more participants in the MFT group (46%) had clinically meaningful changes in pain intensity from T0 to T1 (*p* = 0.012) than the stimulated MFT group (13%). 

## 4. Discussion

This study aimed to evaluate the immediate effect of a standardized MFT on TLF and lumbar erector spinae muscle stiffness in adults with chronic LBP. The secondary objectives were to evaluate the effect of the technique on the thickness of the TLF and pain intensity. To our knowledge, this is the first study to demonstrate signs of stiffness and thickness changes in patients with LBP following the application of an MFT in comparison to a simulated MFT using state-of-the-art imaging technology.

### 4.1. Tissue Stiffness

The main hypothesis of the present study was that the MFT would lead to an immediate decrease in TLF and erector spinae muscle stiffness. Unfortunately, no significant change in TLF stiffness was observed following the application of the MFT when compared to the simulated MFT. In a previous study, Wong et al. reported an immediate decrease in TLF stiffness in 10 asymptomatic adults after a standardized MFT, which was similar to that used in the present study but lasted only three minutes and was applied to one side of the TLF [34]. Various methodological elements may have contributed to the discrepancies between our results and those of the previous study: the small sample size, absence of a control group, population (healthy adults), definition of the outcome variables, and method of measurement. As for the latter, Wong et al. calculated a stiffness index based on the displacement of the fascia and latissimus dorsi muscle junction observed at rest and during muscle contraction with B-mode ultrasonography. The authors suggested that mechanical properties could be more effectively assessed using direct in vivo measurements, such as elastography, as performed in the present study.

Many elements could explain the absence of effects on the fascia in the present study. First, there may be a lack of power given that the sample size was calculated using muscle stiffness, as no studies had been published on changes in TLF stiffness prior to the beginning of our work. Second, it is possible that the dosage of the intervention was not optimal: (1) the load applied may have been inadequate to induce creep or (2) the duration/number of repetitions may have been insufficient. In vitro studies have reported that the change in stiffness is dependent on the strain applied as well as the duration of the strain and the resting period after the application of the strain [51,52].

Based on the results of the present study, the potential of MFTs to reduce the stiffness of the fasciae could also be questioned. In vitro studies have already shown that the strain applied to human and mouse TLFs results in an increase in stiffness after a rest period of 30–60 min [51,52]. Schleip et al. proposed an interesting hypothesis that could possibly explain the perception of tissue change that has been anecdotally reported by manual therapists during MFTs [52]. In Schleip et al.’s study, a decrease in fluid content in porcine TLF was observed during isometric stretching in vitro, with an immediate decrease in stiffness. This was followed by a progressive rehydration of the tissue when the strain was removed, as well as an increase in stiffness, which returned the tissue to its initial state [52]. Therefore, the reduced stiffness felt during palpation might be due to the initial decrease in stiffness that follows the reduction in tissue water content. Nevertheless, these were in vitro studies, one of which used animal tissues. Furthermore, no information about the states of the tissues were presented in these studies (healthy vs. with inflammation). Therefore, the results and interpretations should be considered with caution. Moreover, in the present study, the evaluation of stiffness was performed five minutes after the intervention, which could explain the lack of significant results.

Although there was no effect on TLF stiffness, a significant decrease in erector spinae muscle stiffness was observed in the MFT group compared with the simulated group, which is consistent with the results of previous studies [35,36,37]. In the present study, a 23% decrease in stiffness was observed in the intervention group; this is particularly interesting, as in the same period, the control group showed a 9% increase in stiffness. Interestingly, the change occurred only on the left side, which is the side that was treated first in all participants. This suggests that resting time may be needed after the intervention for the effects to take place in muscle.

The only other study that used SWE to assess muscle stiffness evaluated the iliocostalis muscles following an osteopathic manipulative treatment of the lumbar region, which included several types of manual techniques [35]. Therefore, it is difficult to determine the specific contribution of the myofascial techniques on the immediate reduction in stiffness reported in this study. In the other studies [36,37], differences in the measurement methods used to assess stiffness, the type of techniques used, the dosages, as well as the interventions performed in the control group limit the possibilities of comparison with our results. Differences in the type of technique and dosage are major issues in myofascial studies. There are a variety of different techniques described in the literature, and the load applied to the tissue can vary widely [53]. It is well known that a greater load on the tissue implies a greater deformation; thus, this potentially addresses different mechanisms of action and explains why differences in effects may be observed. The dosage can be interpreted as the duration of the technique, number of techniques applied, and number of treatments. For example, Tamartash et al. performed four sessions of myofascial release on the lumbar region over a two-week period [37], while Lohr and Médina-Porqueres applied two techniques over six minutes [36], including one that involved deep tissue pressure.

### 4.2. Tissue Thickness

A significant difference in thickness in the left TLF was observed between groups, although the difference was very small, with a 1% decrease in the MFT group and a 3% increase in the control group. The decrease in TLF thickness occurred on the same side as the reduction in muscle stiffness. One of the unexpected effects of the technique was the change observed in subcutaneous tissue thickness. The MFT group showed an increase in subcutaneous tissue thickness ranging from 9 to 12%, whereas the simulated group showed a decrease of less than 1%. To our knowledge, the only other study that reported structural changes in fasciae focused on the cervical fasciae [32]. Stecco et al. found a reduction in the thickness of the loose connective tissue between the dense layers of the fasciae, suggesting a change in hyaluronan viscosity [32]. It is difficult to compare our results, as this study addressed the cervical fasciae using Fascial Manipulation^®^, which is a technique that uses stronger tissue pressure than the MFT used in the present study.

The most plausible explanation for the changes observed in tissue thickness is that the strain applied to the tissue during the technique may have induced a movement of fluids as previously described, which would support the results observed in animal tissue in vitro [52]. It seems plausible that the changes in thickness seen in this study may be in part the result of fluid movement between the TLF and the subcutaneous tissue. Another explanation could be that the technique influenced the retinacula cutis, which is a network of vertical and oblique bands of connective tissue connecting the dermis to the superficial fasciae and the superficial fasciae to the deep fasciae [48]. The load induced by the MFT could have led to a deformation of the retinacula cutis followed by relaxation and elongation, resulting in an increase in the thickness of the subcutaneous tissue. The last possibility is that the MFT had an influence on circulation. A recent study showed that the superficial fascia inside the subcutaneous tissue has considerable sensory and sympathetic autonomic innervation [54]. The fibers are present inside the connective tissue, particularly around the blood vessels [54]. These autonomic fibers may play a role in the regulation of the vascularization of the superficial fascia [54]. It is then plausible that the MFT had an effect on the autonomic system, thereby influencing local circulation.

### 4.3. Pain Intensity

The MFT group showed a significant decrease in pain intensity immediately after the intervention and 24 h later when compared to the simulated group, which did not show any changes. This improvement in the MFT group was not sustained over time, as the decrease in pain intensity was not significant seven days post-intervention when compared to the simulated MFT group, even though the values were still below the pre-treatment values. This was predictable given that only one technique was performed over a short period of time in participants suffering from chronic pain for more than 40 months. In this context, it is interesting to note that 46% of the participants in the intervention group had a reduction in pain intensity that reached clinical significance [49]. This threshold was reached even if the pain intensity was low in the pre-treatment measures in the sample. Thus, the intervention provided temporary pain relief, and this favorable effect supports further studies on this type of intervention. It is possible that the change in muscle stiffness led to a decrease in the activation of the enclosed nociceptive fibers. It is also possible that the decrease in muscle stiffness led to a decrease in the tension that the muscle exerts on the fascia when it contracts, leading to a decrease in the stimulation of the nociceptive fibers in the TLF and, therefore, a reduction in pain. Our results regarding pain are in line with those described in the meta-analysis by Wu et al., which included eight randomized controlled trials (n = 375) and concluded that myofascial interventions led to an improvement in pain in patients with chronic LBP [31].

### 4.4. Limitations

Some limitations of the present study should be acknowledged. First, the small sample size is a major limitation. In addition to the limited power that was already discussed, the small sample size resulted in the non-equivalence of groups at baseline for the left muscle stiffness outcome, despite randomization. However, appropriate statistical methods were used to address this issue.

The position of the participants during data collection could also have influenced the results. For some, the prone position was comfortable, while others experienced increased pain despite the care that was taken in the positioning prior to data collection. Some participants had to reposition themselves because of discomfort, which may have increased stiffness in the lumbar region. However, this standard position was necessary for the ultrasound evaluation. In addition, corpulent patients remained in the prone position for a longer period, as it often took several attempts to obtain clear ultrasound images, which could have positively or negatively affected the results.

Another element that could have influenced the results is the measurement instrument. Although sonoelastography provided quantitative data in an area that has not yet been well studied, this instrument is operator-dependent [43]. Thus, the positioning of the probe, its angulation during image capture, as well as the pressure exerted by the evaluator can influence the results [43]. To account for this, and to ensure optimal data collection, we standardized the location for probe positioning and we selected an evaluator with extensive experience in musculoskeletal ultrasound. She performed all image acquisitions according to a standardized evaluation procedure that took into account limitations related to the sensitivity of the instrument. She was also blinded to the intervention received by the participants.

The standardization of the MFT may have also influenced the results. The participants were treated for eight minutes regardless of their initial tissue state. In clinical practice, the dosage of the intervention is normally adapted to the needs of each patient and the tension felt with palpation. However, we chose to conduct a mechanistic study to explore the specific effects of an MFT. Thus, the standardization of the intervention was essential. Although the duration of the strain applied was standardized, the magnitude of the strain and the rate at which it was applied were not controlled. The forces applied to strain the TLF during an MFT are multiaxial. In addition to the compressive force (postero-anterior axis), forces are concomitantly applied in other axes and at different depths to address TLF tensions originating from different directions. The TLF is at the crossroads of many muscles that can influence its tension, including the latissimus dorsi, posterior inferior serratus, obliques, transversus abdominis, and gluteus maximus [22]. The tensile forces of these muscles are exerted on the TLF in different directions depending on their orientation and depth. The magnitude and the direction of the force applied during the technique are challenging to assess. The tissue state also varies among patients. For these reasons, although we recognize the importance of standardizing the force intensity, direction, and rate of application, this could not be applied in the present study, and we instead chose a pragmatic approach corresponding to clinical practice.

Another element that limits the scope of our results is the fact that the participants were evaluated immediately after the intervention (after a 5 min rest period). It would have been relevant to also evaluate the participants 30 or 60 min later to observe the evolution of stiffness, considering the previously discussed in vitro results [51,52]. One of the constraints leading to the choice of assessment time was the potential increase in pain in participants with LBP left in the prone position for a longer period of time.

Finally, it should be noted that the pain assessment was not performed by a blinded assessor. This choice was made to support the study’s feasibility, as pain was a secondary variable; this was considered reasonable as the study included a control group.

Future studies should consider the dosage of the intervention to shed light on (1) the load required to observe a change in the structure and biomechanical properties of the tissues; (2) the optimal duration of the technique’s application; (3) the number of treatments needed to obtain significant changes both in the fasciae and muscle properties, as well as to reach long-term physiological changes in terms of pain modulation. The duration of rest needed after the intervention before a change can take place in the tissue should also be investigated. For pain modulation, future studies should investigate the effect of MFTs in a context closer to clinical reality, including an intervention over several weeks integrating a biopsychosocial approach.

## 5. Conclusions

Non-specific LBP is a condition with a multifactorial etiology. Fasciae are increasingly suspected to be involved in the problem. The purpose of this study was to explore the specific effects of an MFT on the stiffness and thickness of the myofascial tissue and pain intensity of patients with chronic non-specific LBP. An immediate decrease in erector spinae muscle stiffness was observed in the MFT group when compared to the simulated MFT group, as well as a decrease in TLF thickness and an increase in subcutaneous tissue thickness. An immediate and short-term decrease in pain intensity was also observed in the MFT group in comparison to the simulated group. This study is one of the first to objectively validate the effect of myofascial techniques with shear wave sonoelastography.

## Figures and Tables

**Table 1:** Characteristics of participants in simulated and MFT groups.

Parameters	Sample (n = 48)	MFT (n = 24)	Simulated MFT (n = 24)	*p* Value
Age (years), mean ± SD	37.4 ± 13.3	37.9 ± 14.2	37.0 ± 12.7	0.81 ^a^
Sex (male/female), n, %	24/24 (50%/50%)	12/12 (50%/50%)	12/12 (50%/50%)	1.00 ^b^
Dominance (right/left), n, %	46/2 (91.7%/8.3%)	24/0 (100%/0%)	22/2 (91.7%/8.3%)	0.49 ^c^
Duration of pain (months), median (IQR 25th–75th)	48 (36–69)	42 (15–60)	60 (42–90)	0.09 ^d^
BMI (kg/cm^2^), median (IQR 25th–75th)	24.5 (22.6–29.6)	24.7 (22.4–30)	24.3 (22.6–28.5)	0.89 ^d^
Schober index (cm), mean ± SD	6.8 ± 1.6	6.5 ± 1.6	7.1 ± 1.5	0.18 ^a^
SF-MPQ total score (/45), median (IQR 25th–75th)	11 (9–15.8)	11 (10–14)	11 (8.3–17.8)	0.95 ^d^
SF-MPQ sensory score (/33), median (IQR 25th–75th)	9 (7.3–12)	8 (7–13.5)	8 (7–13.5)	0.72 ^d^
SF-MPQ affective score (/12), median (IQR, 25th–75th)	2 (1–3)	2 (1–3)	2 (1–3)	0.69 ^d^
SF-MPQ VAS (cm), mean ± SD	44.0 ± 19.1	49.3 ± 19.4	38.7 ± 17.6	0.05 ^a^
SF-MPQ present pain index (0–5), n, %	1:18 (37.5%)	1:7 (29.2%)	1:11 (45.8%)	0.16 ^d^
	2:23 (47.9%)	2:12 (50%)	2:11 (45.3%)	
	3:5 (10.4%)	3:4 (16.7%)	3:1 (4.2%)	
	4:1 (2.1%)	4:0	4:1 (4.2%)	
	5:1 (2.1%)	5:1 (4.17%)	5:0	
ODS (%), mean ± SD	21.4 ± 8.5	21.4 ± 9.3	21.3 ± 7.8	0.97 ^a^
IPAQ (MET/week), median (IQR 25th–75th)	3197.8 (1575–6004)	2435 (1599–6975)	3496 (1502–5220)	0.78 ^d^
	(n = 46)	(n = 23)	(n = 23)	
IPAQ categorical score (low/moderate/high), n, %	Low: 8 (17.4%)	Low: 4 (17.4%)	Low: 4 (17.4%)	1.00 ^d^
	Mod: 10 (21.7%)	Mod: 5 (21.7%)	Mod: 5 (21.7%)	
	High: 28 (60.9%)	High: 14 (60.9%)	High: 14 (60.9%)	
	(n = 46)	(n = 23)	(n = 23)	
IPAQ sitting (hours/week)	39.2 ± 18.3	39.7 ± 17.6	38.6 ± 19.4	0.83 ^a^

Note: BMI—body mass index; SF-MPQ—Short-form McGill Pain Questionnaire; VAS—visual analog scale; ODS—Oswestry Disability Scale; IPAQ—International Physical Activity Questionnaire; ^a^ t test, independent samples; ^b^ Chi-squared test; ^c^ Fisher’s exact test; ^d^ Mann–Whitney U test, independent samples.

**Table 2:** Stiffness assessed with SWE (in kPa) at each time point and differences between treatment groups.

Structures	Descriptive Statistics						Generalized Estimating Equation Models			
	MFT			Simulated MFT			Mean Difference between Groups	95% CI		*p* Value
	n	Mean	(SD)	n	Mean	(SD)				
TFL-R										
Pre-treatment	24	75.3	37.2	24	59.6	24.8				
Post-treatment	24	74.4	37.4	23	71.6	35.4	−13.0	−32.5	6.5	0.191
TFL-L										
Pre-treatment	24	73.4	38.6	24	60.6	35.3				
Post-treatment	24	70.9	40.0	23	59.3	17.5	−1.2	−17.7	15.2	0.880
Muscle-R										
Pre-treatment	24	52.5	32.7	22	44.1	25.1				
Post-treatment	24	50.6	42.4	21	51.6	35.5	−9.2	−26.4	7.9	0.290
Muscle-L										
Pre-treatment	23	54.0	37.8	23	39.5	18.2				
Post-treatment	23	41.5	17.7	22	43.1	14.6	−16.1	−31.1	−1.14	0.035 *

Note. SD—standard deviation; CI—confidence interval; TLF—thoracolumbar fascia; R—right; L—left; * significant value.

**Table 3:** Thickness (in mm) assessed with ultrasound at each time point and differences between treatment groups.

Structures	Descriptive Statistics						Generalized Estimating Equation Models			
	MFT			Simulated MFT			Mean Difference between Groups	95% CI		*p* Value
	n	Mean	(SD)	n	Mean	(SD)				
TFL-R										
Pre-treatment	24	2.00	0.64	24	1.80	0.45				
Post-treatment	24	1.95	0.62	23	1.77	0.41	−0.02	−0.10	0.06	0.640
TFL-L										
Pre-treatment	24	1.90	0.50	24	1.78	0.42				
Post-treatment	24	1.88	0.52	23	1.83	0.43	−0.07	−0.13	0.00	0.039 *
SC tissue-R										
Pre-treatment	24	10.7	5.55	24	9.62	6.37				
Post-treatment	24	11.8	5.84	23	9.58	6.67	1.15	0.60	1.71	<0.0001 *
SC tissue-L										
Pre-treatment	24	10.4	5.48	24	9.48	6.38				
Post-treatment	24	11.7	5.71	23	9.37	6.39	1.46	0.41	2.52	0.007 *

Note. SD—standard deviation; CI—confidence interval; TLF—thoracolumbar fascia; SC—subcutaneous; R—right; L—left; * significant value.

**Table 4:** Pain intensity measured with the numerical rating scale at each time point and differences between treatment groups.

	Descriptive Statistics						Generalized Estimating Equation Models			
Time	MFT			Simulated MFT			Mean Difference between Groups	95% CI		*p* Value
	n	Mean	(SD)	n	Mean	(SD)				
Pain										
Pre-treatment (T0)	24	3.3	1.6	24	3.0	1.5				
Post-treatment (T1)	24	1.9	1.7	24	2.9	1.6	−1.3	−2.1	0.6	<0.0001 *
Post-treatment (T2)	24	2.3	1.6	24	2.7	1.3	−0.8	−1.5	0.0	0.043 *
Post-treatment (T7)	24	2.5	1.5	24	3.1	1.7	−0.9	−1.9	0.1	0.081

Note. SD—standard deviation; CI—confidence interval; * significant value.

## Data Availability

The data presented in this study are available upon request from the corresponding author.
